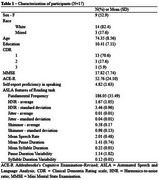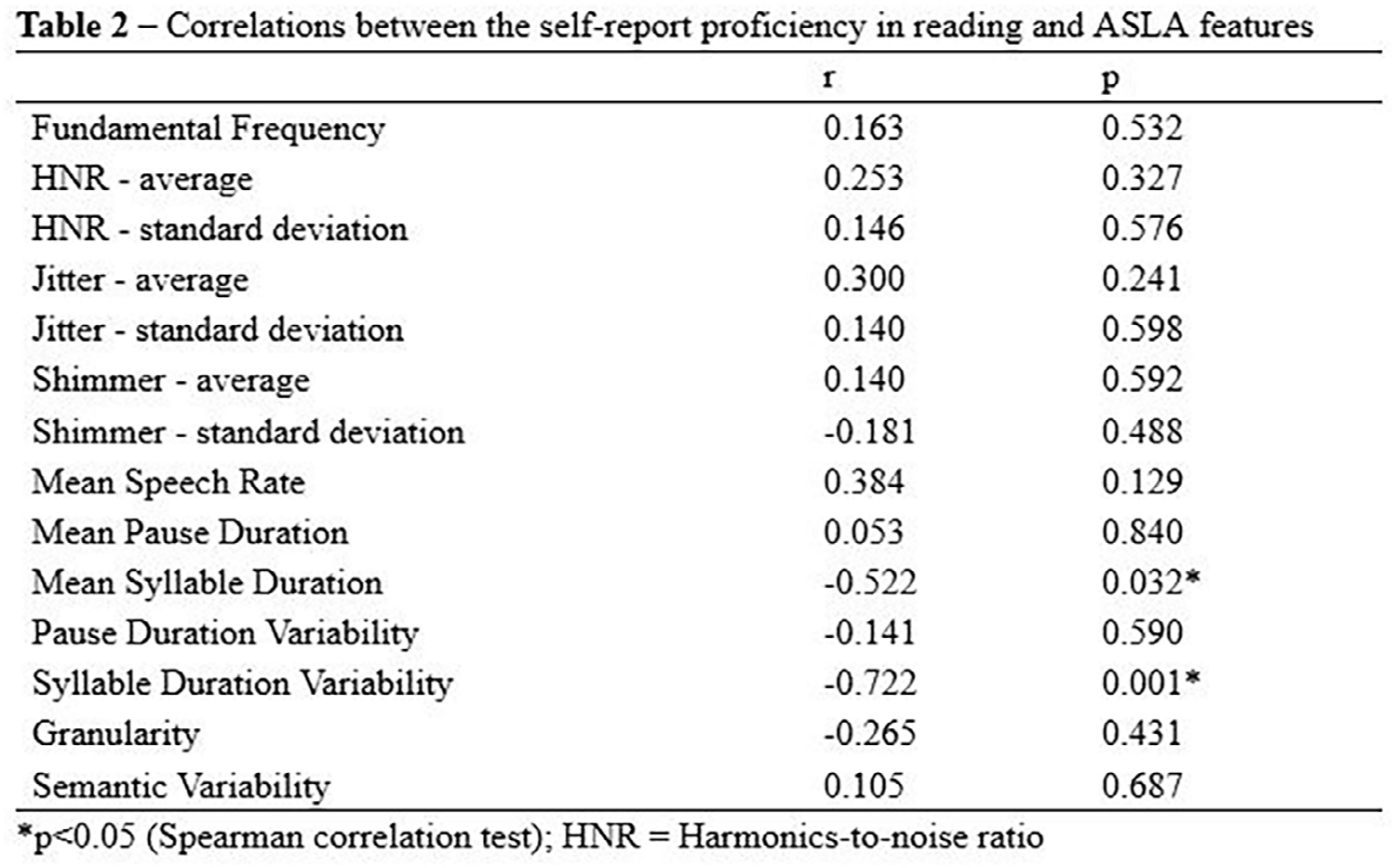# Preliminary correlations between objective and self‐reported speech measures in Portuguese‐speaking Alzheimer's disease patients

**DOI:** 10.1002/alz70860_100199

**Published:** 2025-12-23

**Authors:** Guilherme Briczinski Souza, Daniel Coswig Zitzke, Gabriela Sanches, André Luiz Rodrigues Palmeira, Liana Lisboa Fernandez, Adolfo M Garcia, Bárbara Costa Beber

**Affiliations:** ^1^ Universidade Federal de Ciências da Saúde de Porto Alegre (UFCSPA), Porto Alegre, Rio Grande do Sul, Brazil; ^2^ Global Brain Health Institute (GBHI), University of California San Francisco (UCSF); & Trinity College Dublin, Dublin, Ireland; ^3^ Cognitive Neuroscience Center, University of San Andrés, Victoria, Buenos Aires, Argentina; ^4^ Universidad de Santiago de Chile, Santiago, Santiago, Chile

## Abstract

**Background:**

Alzheimer's disease (AD) typically involves alterations in speech and language production. These are often obvious to patients themselves, but no study has assessed whether self‐reported estimations of speech difficulties reflect objective variations in speech parameters. This exploratory study tackles this gap by testing correlations between self‐reported speech ratings and automated speech measures in a sample of Portuguese‐speaking Brazilians with AD.

**Method:**

Between March and December 2024, data were collected from individuals meeting the NINCDS‐ADRDA diagnostic criteria for AD. Patients self‐reported their speaking proficiency and perceived difficulties via an adaptation and extension of the Bilingual Language Profile [3,4], using a Likert scale that ranged from 0 to 6, where higher values represent greater proficiency. Spontaneous speech samples were collected through a routine description task on the Toolkit to Examine Lifelike Language (TELL), a validated speech biomarker app. We targeted variables related to voice quality and speech timing (Table 1). Associations between TELL variables and self‐report scores were tested using Spearman's correlations, with a significance level of 0.05.

**Result:**

The sample comprised 17 AD individuals, with a mean self‐reported speaking proficiency of 4.82 (±1.63) (Table 1). A significant negative correlation was found between self‐reported speaking proficiency and both mean syllable duration (*r* = ‐0.522, *p* = 0.032) and syllable duration variability (*r* = ‐0.722, *p* = 0.001) (Table 2).

**Conclusion:**

These findings suggest self‐perceived speaking abilities in AD are reliably related to syllable duration. Though preliminary, such results suggest that subjective impressions of language production may be related to objective speech patterns, paving the way for reappraisals of patients’ own judgments of their verbal skills.